# Supplementary material for: NSC828779 Alleviates Renal Tubulointerstitial Lesions Involving Interleukin-36 Signaling in Mice
Source: Cells. 2021 Nov 6;10(11):3060. doi: 10.3390/cells10113060 (PMC8623783; doi:10.3390/cells10113060)
Supplement: Supplementary file 1 [file cells-10-03060-s001.zip › cells-1427395-supplementary.pdf]

Supplemental Table

Table S1. ***In-silico*** **molecular docking analysis with NSC828779 and standard STAT3 inhibitor**

Ligand	Receptor	PDB ID	Binding affinity (kcal/mol)	Bonding length (Å)	Amino acid residue	Interactions
NSC828779	STAT3	4ZIA	-9.2	2.63	SER-113	Conventional hydrogen bond
				3.76	TRP-43	Carbon Hydrogen Bond
				3.11	SER-48	Carbon Hydrogen Bond
				3.35	ALA-44	Halogen (Fluorine)
				3.37	ALA-47	Halogen (Fluorine)
				3.22	ALA-106	Halogen (Fluorine)
				3.47	GLN-41	Halogen (Fluorine)
				4.07	TRP-110	Pi-Pi Stacked
SH-4-54	STAT3	4ZIA	-8.4	2.03	ALA-44	Conventional hydrogen bond
				2.36	SER-113	Conventional hydrogen bond
				3.61	TRP-110	Carbon hydrogen bond
				3.57	ARG-114	Carbon hydrogen bond
				3.05	ALA-106	Halogen (Fluorine)
				3.48	TRP-43	Halogen (Fluorine)

Analysis of the interactions between NSC828779 and the receptor revealed a higher number of halogen bonding, conventional H-bonding, carbon-hydrogen bonding, and pi interactions created on the ligand backbone with a higher number of amino acids residues than SH-4-54 in STAT3 cavity. However, the overall binding affinity of STAT3 with a known inhibitor, SH-4-54, was less negative than the value obtained for NSC828779-STAT3 complex.

Supplementary Figure Legends

Figure S1. NSC828779 alleviates pathological changes in UUO mice. (A) IHC for Col-I. (B) Scoring of Col-I positive area. Original magnification, each 400×. NSC828779, a salicylanilide derivative. UUO, unilateral ureter obstruction; TILs, tubulointerstitial lesions. Col-I, Collagen I. Data are shown as means ± SEM of seven mice per group. The horizonal dashed line indicates the mean for sham control+NSC828779. The bar indicates 20 μm. Arrows indicate positive staining. $^{**}p < 0.01$, $^{****}p < 0.001$. ‡no significant difference Day 7 versus Day14 of UUO+NSC828779.

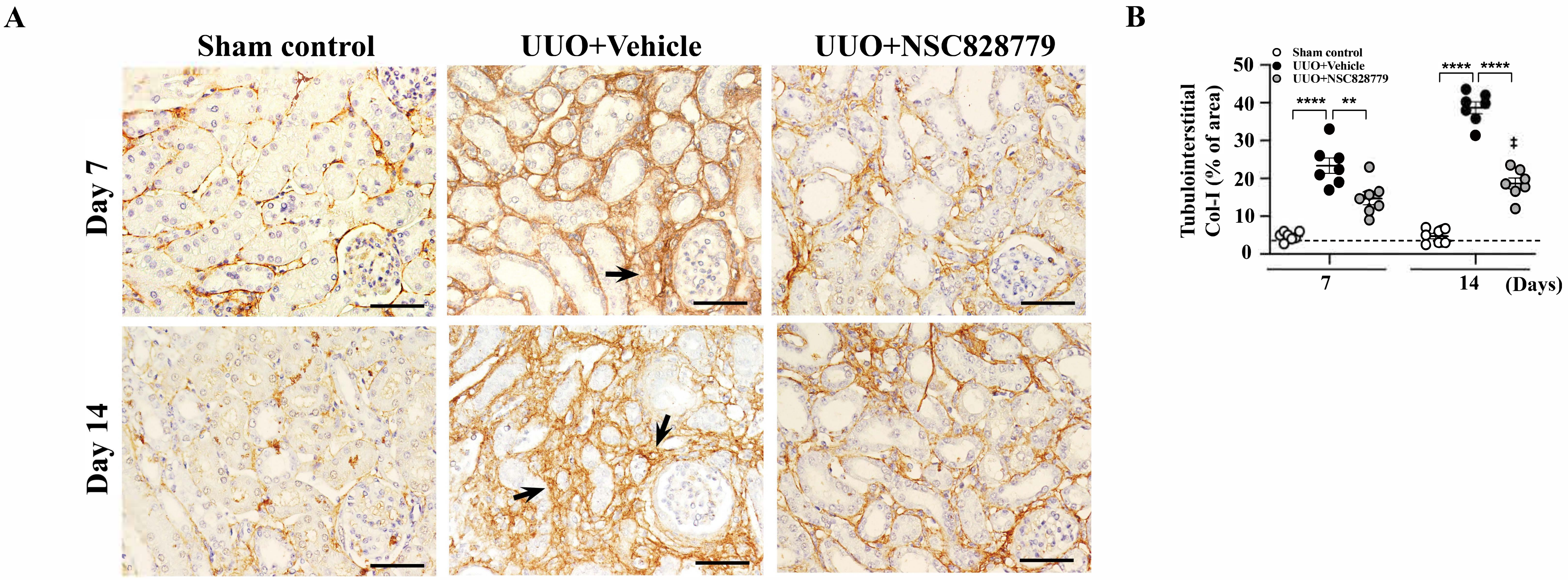

Supplementary Figure S1. Yang *et al.*
